# Follow-Up of Side Effects throughout the Entire Course of Coronavirus Vaccination

**DOI:** 10.3390/vaccines11030704

**Published:** 2023-03-20

**Authors:** Mohanad Odeh, Ghada Nazar Al-Jussani, Abdelrahman Ashour, Husam AlNaqah, Hamza A. Hasan, Lana Sbitan, Amro Dawabsheh, Moayad Alhawi

**Affiliations:** 1 Department of Clinical Pharmacy and Pharmacy Practice, Faculty of Pharmaceutical Sciences, The Hashemite University, P.O. Box 330127, Zarqa 13133, Jordan; 2 Department of Pathology and Forensic Medicine, Faculty of Medicines, Hashemite University, P.O. Box 330127, Zarqa 13133, Jordan; 3 Faculty of Medicine, Hashemite University, P.O. Box 330127, Zarqa 13133, Jordan

**Keywords:** side effects, COVID-19, vaccines, Jordan, pharmacovigilance

## Abstract

Vaccines are considered the best protective means against coronavirus infection. There is increasing interest in reporting the side effects of vaccines, especially for individuals younger than 18 years old. Accordingly, this analytical cohort study aims to report on the side effects of adult and young individuals who received vaccination within 24 h, 72 h, 5 days, and 1 week through the entire course of vaccination (ECoV). A validated online survey was used to collect information. In total, 1069 individuals were completely followed. Most individuals received the Pfizer vaccine (59.6%). Most individuals had received two doses (69.4%). Very strong and statistically significant associations with side effects (*p* < 0.05, Phi (Φ) > 0.25) throughout the ECoV were reported for the type of vaccine and female gender. Non-smokers reported weak statistically significant associations. Fatigue and localized pain were the most commonly reported side effect, with onset within 24 h and duration of less than 72 h. The prevalence of reported side effects was statistically significantly higher among young individuals (<18 years old) than among adults (X^2^ (1) =7.6, *p* = 0.006. Phi φ = 0.11).

## 1. Introduction

Coronavirus disease 2019 (COVID-19) is an infectious disease caused by severe acute respiratory syndrome coronavirus 2 (SARS-CoV-2), which mainly affects the respiratory system [1]. The first recorded case was in Wuhan, China, in December 2019 [2]. The World Health Organization (WHO) declared COVID-19 a pandemic in March 2020 [2]. Thereafter, developing a vaccine became a high priority to confront this pandemic. Pfizer-BioNTech and Oxford-AstraZeneca are nucleic acid vaccines based on messenger ribonucleic acid (mRNA) and double-stranded deoxyribonucleic acid (dsDNA), respectively [3]. Sinopharm, on the other hand, is an inactivated coronavirus vaccine [4].

In January 2021, the Jordanian ministry of health launched the national vaccination program, which was set to include all children over 12 years old in the vaccination process. The most used vaccines in Jordan are Pfizer-BioNTech, Sinopharm, and Oxford-AstraZeneca. In July 2022, more than four million adults had been vaccinated with two doses, while more than 500,000 had received three doses [5].

Despite the efficacy of COVID-19 vaccines, there are many common side effects [6,7]. The incidence may differ greatly depending on the type of given vaccine. Sinopharm has shown the mildest side effects, such as pain at the injection site, fatigue, headache, lethargy, and muscle pain. In rare situations, however, vaccine-induced immune thrombotic thrombocytopenia (VITT) has been recorded as a life-threatening side effect [8,9]. The Oxford-AstraZeneca vaccine has shown comparatively more serious side effects, which include thrombosis, Guillain–Barré syndrome, acute transverse myelitis, myocarditis, pericarditis, and glomerular disease [6]. 

The side effects of the Pfizer-BioNTech vaccine are most commonly mild to moderate and include muscle pain, joint pain, itching, lymph node swelling, nausea, dyspnea, and diarrhea, with a minor incidence of life-threatening side effects such as angioedema, pancarditis, Bell’s palsy, and anaphylactic shock [10].

Notwithstanding the fact that adolescents and children aged below 18 years mostly exhibit less severe SARS-CoV-2 infection than adults, severe symptoms have also been reported in these age groups, specifically in individuals with underlying medical comorbidities [11]. Attributable to their role in SARS-CoV-2 transmission, vaccination of adolescents and children may contribute to the development of herd immunity within societies [12,13]. The pandemic has impacted in-person learning, socialization, and ultimately, the education of school-aged children and adolescents [14,15]. 

The Food and Drug Administration expanded the emergency use authorization for the BNT162b2 (Pfizer-BioNTech) vaccine on 10 May 2021, including individuals 12 years of age or older [16], which made it the only vaccine authorized for use in adolescents younger than 16 years of age. In a recent study titled “Safety, Immunogenicity, and Efficacy of the BNT162b2 COVID-19 Vaccine in Adolescents”, BNT162b2 was found to have high efficacy against SARS-CoV-2 in participants aged 12 to 15 years and elicit a more significant immune response with a favorable safety profile, mostly ephemeral mild-to-moderate side effects (primarily, injection site pain, fatigue, and headache) [17]. Severe adverse events following the administration of BNT162b2, including myocarditis, are scarcely reported in adolescents [18].

Most of the adverse effects identified in adults are reported as mild-to-moderate events for which no in-patient treatment is required [19]. Severe adverse events have been outlined, most requiring hospitalization and possibly life-threatening, including venous thromboembolism, stroke, myocardial infarction, pulmonary embolism, myocarditis, and pericarditis [17,20,21,22,23].

There is still a need to explore side effects reported for individuals who have received vaccines along the entire course of vaccination (ECoV), including those who are less than 18 years old. The present research was therefore designed as an analytical cohort study to report the side effects of adult and young individuals who received vaccination within 24 h, 72 h, 5 days, and 1 week throughout the ECoV. We then explored the associations and measured the nominal-by-nominal relationship between independent variables and the reported side effects during the ECoV.

## 2. Materials and Methods

A longitudinal analytical cohort study. An online platform was developed to collect data, with six well-trained medical students collecting data from the vaccination center at Hashemite University and another two centers from 15 November 2021 to 30 August 2022. Data were collected for those who agreed to take part in the study face-to-face while visiting the center and then by phone call using their registered mobile phone numbers 1 day, 3 days, 5 days, and 1 week after each injection. Accordingly, those who received a single dose of the vaccine were contacted after injection. Those who received two doses were contacted after the first and second shots of the vaccine. Those who received three doses were contacted after the first, second, and third shots. Those who received heterogeneous vaccinations were excluded from the study. 

The Google Sheets platform was used to develop a spreadsheet to collect data from participants, with a specific ID assigned for each. In the process of designing the questions, the research team shared the draft with eight experts, who provided comments and feedbackthat were considered by the research team. Only items that had an accepted content validity ratio (CVR) were used, i.e., with the assessment of eight experts, only items with Lawshe’s content validity ratio (CVR) > 0.741, *p* < 0.05 were considered [24].

The questionnaire had the following sections: (1) demographics; (2) comorbidities, allergies, and medication; (3) the profile of side effects (symptoms of the side effect, onset, duration, severity—impact on daily life, data for which were collected after each injection throughout the ECoV). The categories for side effects [19] were classified as mild (i.e., does not interfere with daily activity such as general tasks, mood, mobility, and normal work), moderate (i.e., interferes with daily activity, but no hospitalization), or severe (cases involving hospitalization). 

Before enrollment, participants received a full description of the study, they were informed that their vaccination number would be recorded for follow-up and would be used as their ID number. No specific personal data were collected, and data would be treated as confidential in all cases. Subjects were also informed that participation is totally voluntary, and they could withdraw at any stage without the need to clarify reasons for their request. The study protocol was approved by the institutional review board (IRB) committee at Hashemite University (No. 13/1/2021/2022).

In Jordan, almost 3.53 million people were fully vaccinated by Nov 2021 [5,25]. Based on this number, a sample size of minimum of 385 participants was considered representative as recommended by Taherdoost [26] and carried out by the sample size calculator [27] considering a confidence level of 95% and a margin of error of 5%.

Data were coded and transferred to SPSS for Windows, version 25, for statistical analysis (SPSS Inc., Chicago, IL, USA). For data analysis, descriptive analysis was first conducted to present participant characteristics. The chi-squared test (X^2^) for association was used (Fisher’s exact test was used in cases where the expected count was less than 5). To measure the strength of the association of a nominal-by-nominal relationship (which is a measure of effect size), the Phi (Φ) coefficient factor was also calculated and illustrated, with a strength of association value of 1 indicating complete association, “0” indicating no association, >0.25 indicating a very strong relationship, >0.15 indicating a strong relationship, >0.1 indicating a moderate relationship, and >0.05 indicating a weak relationship [28]. The degrees of freedom for the chi-square between practices were illustrated using the formula df = (r − 1)(c − 1), where r is the number of categories within the demographic variable and c is the number of options related to item options. For all tests in the present study, a *p*-value of <0.05 is considered to indicate a statistically significant difference [29].

## 3. Results

Results are presented within the following sections: Section 3.1 Descriptive results for the participant and comorbidities. Section 3.2 Descriptive for side effect types, onset, duration, and impact. Section 3.3 Analysis of the relationship and association within and between variables.

### 3.1. Descriptive Results

#### 3.1.1. Descriptive Results for Individuals

Table 1 shows the characteristics of the 1069 individuals who were enrolled in our study. In all, 542 (50.7%) were male and 527 (49.3%) were female. The majority were young, between 18 and 40 years old (63.7%), living in Amman (701, 65.6%), and non-smokers (874, 81.8%). Regarding the highest educational level, 685 (64.1%) of participants held a bachelor’s degree, 104 (9.7%) held a diploma degree, 49 (4.6%) held a master’s/PhD degree, 52 (4.9%) had attended primary school, and 179 (16.7%) had attended secondary school. Most participants were non-HCWs (662 or 62% compared with 406 or 38% who worked in healthcare). The majority of participants received the Pfizer-BioNTech vaccine (637, 59.6%), followed by Sinopharm (387, 36.2%) and AstraZeneca (45, 4.2%).

#### 3.1.2. Description of Comorbidities and Other Variables (Allergies and Medications)

Table 2 shows that spring (seasonal) allergies were the most common, affecting 25.26% of total participants, with the least common allergy found to be walnut fruit allergy. Among chronic diseases, hypertension was the most common, followed by diabetes and coronary artery disease, while only eight individuals reported liver disease and neurological disease. The most common medication type was painkiller (NSAID and others), followed by hypertension and anti-coagulant.

### 3.2. Descriptive for Side Effects Types, Onset, Duration, and Impact

#### 3.2.1. Type of Side Effects and Number of Doses

Table 3 shows the incidence of side effects according to the number of doses that the individual received. The majority of individuals suffer from fatigue, fever, headache, localized pain, and myalgia. Other individuals suffered from arthralgia, chills, and localized redness. A small minority of individuals suffered from side effects such as dizziness, diarrhea, cough, rhinorrhea, nasal congestion, sneezing, shortness of breath, nausea, vomiting, sore throat, facial swelling, ageusia, anosmia, and neurological symptoms.

#### 3.2.2. Severity of Reported Side Effects

**Figure 1:**
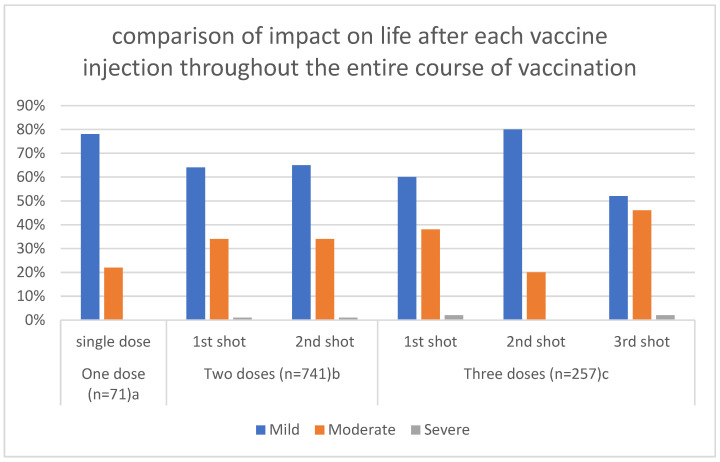
The severity of side effects after each vaccine injection throughout the entire course of vaccination. a: Pfizer-BioNTech, *n* = 46 (64.8%), Sinopharm, *n* = 21 (29.6%), AstraZeneca, *n* = 4 (5.6%). b: Pfizer-BioNTech, *n* = 410 (55.3%), Sinopharm, *n* = 303 (40.9%), AstraZeneca, *n* = 28 (3.8%). c: Pfizer-BioNTech, *n* = 181 (70.4%), Sinopharm, *n* = 63 (24.5%), AstraZeneca, *n* = 13 (5.1%).

Most individuals suffer side effects with mild impact, with less than 3% requiring hospitalization for complications (Figure 1).

#### 3.2.3. Side Effects and 1-Week Follow-Up

The majority of individuals suffered from side effects within 24 h of vaccination. Other individuals suffered from side effects 1–3 days after vaccination. A small minority of individuals suffered from side effects later than 1 week after vaccination. As seen in Table 4, almost 14% of those who received three doses suffered from minor side effects for periods longer than 1 week.

### 3.3. Analysis of the Relationship, Associations within and between Variables

Table 5 shows the frequency of reported side effects and their association with different variables after each injection of vaccine throughout the entire course of vaccination.

#### 3.3.1. Individuals Who Received a Single Dose

The profiles of side effects show no statistically significant differences across all tested variables (gender, age, smoking status, and vaccines).

#### 3.3.2. Individuals Who Received Two Doses

After the first shot for those who received two doses of vaccines, individuals receiving the Pfizer vaccine reported a statistically significant higher percentage of side effects compared with those receiving Sinopharm and AstraZeneca vaccines (64.7% vs. 28.8% and 6.5%, respectively, X^2^ (4) = 50.8, *p* < 0.001), and the association was very strong (Phi φ = 0.26). Females reported statistically significant side effects more than males (57.6% vs. 42.2%, X^2^ (1) = 164.5, *p* < 0.001, and the association was very strong (Phi φ =0.53). Non-smokers reported statistically significant side effects compared with smokers (X^2^ (1) = 4.2, *p* = 0.042); however, the association strength was weak (Phi φ = 0.08). In line with the results from the first shot, variables such as gender, smoking, and the type of vaccine maintained their statistically significant association with the side effect profile. However, at this stage, a new variable “age’’ was also reported to be statistically significantly associated. Patients receiving the Pfizer vaccine reported a statistically significant higher percentage of side effects compared with Sinopharm and AstraZeneca (70.7% vs. 24.3% and 5.1%, respectively, X^2^ (4) = 54.7, *p* < 0.001), and the association was very strong (Phi φ =0.27). Significantly more females than males reported side effects (58% vs. 42%, X^2^ (1) =21.3, *p* < 0.001, and the association was very strong (Phi φ = 0.38). Non-smokers reported a higher percentage of side effects, but the association was weak (Phi φ = 0.08). After the second shot for those who received two doses of vaccines, the prevalence of reported side effects versus no side effects among young individuals (<18 years old) was higher than for the elderly (>18 years), and this difference was statistically significant (X^2^ (1) =7.6, *p* = 0.006, and the association strength was moderate (Phi φ = 0.11.)

#### 3.3.3. Individuals Who Received Three Doses of Vaccine

After the first shot for those who received three doses of vaccines, a statistically significant higher percentage of side effects was reported for patients receiving the Pfizer rather than Sinopharm and AstraZeneca vaccines (76.9% vs. 15.7% and 7.4%, respectively, X^2^ (4) = 16.2, *p* = 0.002), and the association was strong (Phi φ =0.25). Significantly more females than males reported side effects (63.6% vs. 36.4%, X^2^ (1) = 4, *p* = 0.045). However, the strength of the association was weaker than that reported for those who had received two doses (Phi φ = 0.13 vs. Phi φ = 53). 

## 4. Discussion

The present study showed that after the first shot of those who received two doses of vaccines, individuals that received the Pfizer vaccine reported a statistically significant higher percentage of side effects compared with those receiving Sinopharm and AstraZeneca vaccines. This finding contradicts the conclusion of two studies that were conducted to compare different types of vaccines against COVID-19, which found more side effects resulting from the AstraZeneca vaccine compared with Pfizer and Sinopharm vaccines [30,31]. This can be attributed to the lower number of participants who received the AstraZeneca vaccine in our study. 

Similar to the findings of several published studies in Jordan or worldwide [8,31,32,33,34,35,36,37], the most commonly reported side effects following COVID-19 vaccination, regardless of the type of vaccine, are fatigue, fever, headache, localized pain, and myalgia. In the present study, the severity of side effects was assessed by the questionnaire. Most responders (nearly 90%) reported a mild to moderate impact on life, while less than 3% required hospitalization. This is in agreement with similar studies conducted in the same region [8,32,33,38]. Moreover, our study showed that the majority of vaccine recipients had vaccination side effects for less than 3 days’ duration, which is in line with the results of a study conducted in India illustrating that vaccination side effects tend to resolve within 48 hours of vaccine administration [39]. 

There are many studies that demonstrate the variety of vaccine side effects based on gender [40]. The present study shows that females experienced more side effects compared with males [40,41], and the variation between males and females is attributed to sex hormones, including estrogen, progesterone, and testosterone, which can bind to the surface of immune cells and influence how they work. Exposure to estrogen causes immune cells to produce more antibodies in response to the vaccine in general [42]. Some studies reported no difference between males and females [43]. Nevertheless, there are others showing that more males suffer from side effects than females [44]. Our results support those studies and scientific explanations reporting females having a higher sensitivity to side effects. In the present study, differences in side effect profiles based on gender had diminished in those who received three doses of vaccines. 

The present results highlight that non-smokers are more susceptible to experiencing more significant side effects in comparison with smokers. This finding is in agreement with the results of studies by Abukhalil et al. and Hatmal et al. [45,46]. However, in the study of Al-Hanawi et al., a higher prevalence of moderate adverse effects among smokers in contrast to non-smokers was demonstrated [47]. Historically, the influence of smoking on vaccination has been studied. A study conducted in the Netherlands showed a greater response of antibodies toward the influenza vaccine in smokers compared with non-smokers, which might indicate a modulatory role of smoking over the immune system that in turn affects the body’s response toward vaccination [48].

The present study evaluated the implications of the Pfizer-BioNTech vaccine in participants aged <18 years old. After the second shot of those who received two doses of the vaccine, the prevalence of reported side effects versus no side effects was higher among young individuals (<18 years old) than among adults (>18 years old). Such results are similar to those reported in other studies [17,19]. This might be explained by increased reactogenicity and increased vaccine-induced immunogenicity, which may lead to more intense inflammatory responses in younger people [19,49]. Most side effects described after the second dose of the Pfizer-BioNTech vaccine in young participants (<18 years old) are transient mild-to-moderate side effects (predominantly injection site pain, fatigue, headache, fever, and myalgia) as confirmed by Robert and colleagues [17].

Similar to the findings of our study, there is increasing evidence confirming that females and younger people tend to report more side effects from COVID-19 vaccines [50]. Nevertheless, nocebo effects [51,52] of COVID-19 vaccines should be considered [53,54]. The incidence of nocebo response to COVID-19 vaccines was reported to be high (16.4%) in a recent meta-analysis [55]. Earlier systematic reviews found little evidence for a gender effect [56], with females observed to be more vulnerable to nocebo effects [57]. While nocebo effects are driven more by misinformation (verbal manipulation) in males, they are found to be driven by conditioning and previous experience in females [58]. This lagged effect is further documented by Hoffman and colleagues [59], who reported that COVID-19 vaccine side effects decreased across waves for males but increased for females. Accordingly, the association between wave one and wave two side effects (wave one following a second Pfizer dose, wave two after their booster) was more robust in females. They also reported no association across the waves between side effects and age. Such results are strongly linked with the findings of the present study. 

### 4.1. Strengths

This study has numerous strengths. It follows the side effect profile after each vaccine injection despite the number of doses, and information is then presented for each injection and dose of vaccine. Such presentation went hand-in-hand with related statistical analysis. For the present study, the recruited participants amounted to a very robust sample size, especially for individuals younger than 18 years who received the vaccination. 

### 4.2. Limitations

The present study has some limitations, for example, we could not access the hospital admissions after vaccination to validate the causality association between vaccine and admission. Information in the present study is mainly dependent on participants’ self-assessment, which may differ from one person to another. In addition to the standard bias are the participants’ recall bias [60] and the Hawthorne effect [61].

## 5. Conclusions

The profile of side effects in terms of prevalence, severity, onset, and duration of action was almost constant across the entire course of vaccination. Most side effects, regardless of the type of vaccine, were mild and disappeared 3 days after injections. One week after vaccination was the period corresponding to being totally free of side effects. Mainly females and, to a lesser extent, non-smokers were more sensitive to side effects and reported consistent claims across the entire course of vaccination. In the present study, the Pfizer-BioNTech vaccine was the most used vaccine, followed by Sinopharm and AstraZeneca. Individuals younger than 18 years of age reported a higher prevalence of side effects compared with older individuals.

## Figures and Tables

**Table 1:** Characteristics of the participants.

Variable	Outcome		Frequency (*n* = 1069)	Percentage (%)
Gender	Male		542	50.7%
	Female		527	49.3%
Ag	<18		145	13.6%
	18–40		681	63.7%
	40–60		179	16.7%
	More 60		64	6%
Education	Primary School		52	4.9%
	Secondary School		179	16.7%
	Diploma		104	9.7%
	Bachelor’s		685	64.1%
	Higher Education (Master/PhD)		49	4.6%
Occupation	HCWs		406	38%
	Non HCWs		662	62%
Smoking				
Smoker	Yes		195	18.2%
	No		874	81.8%
Duration of Smoking (Years)	<5		85	43.6%
	5–10		48	24.6%
	>10		62	31.7%
Packets (Per day)	1		153	78.4%
	2		38	19.5%
	3 or more		4	2%
Place of Residence	Amman		701	65.6%
	Zarqa		187	17.5%
	Irbid		35	3.3%
	Mafraq		34	3.2%
	Balqa’		28	2.6%
	Jerash		25	2.3%
	Madaba		15	1.4%
	Karak		13	1.2%
	Tafila		12	1.1%
	Ajloun		9	0.8%
	Ma’an		5	0.5%
	Aqaba		5	0.5%
Vaccine type	Pfizer-Biontic		637	59.6%
	Sinopharm		387	36.2%
	AstraZeneca		45	4.2%
Number of doses	Single dose		71	6.6%
	Two doses		741	69.4%
	Three doses		257	24.0%
Vaccine type and doses	Pfizer-Biontic, *n* = 637	Single dose	46	7.2%
		Two doses	410	64.4%
		Three doses	181	28.4%
	Sinopharm, *n* = 387	Single dose	21	5.4%
		Two doses	303	78.3%
		Three doses	63	16.3%
	AstraZeneca, *n* = 45	Single dose	4	8.9%
		Two doses	28	62.2%
		Three doses	13	28.9%

**Table 2:** Allergies, Comorbidities, and Medications for individuals.

Allergy	Yes	No	Not Sure	Total
Bee allergy	34	881	154	1069
Egg allergy	19	1029	21	1069
Fruit allergy	6	1003	60	1069
Milk allergy	32	1005	32	1069
Walnut allergy	8	1037	24	1069
Drug allergy	59	897	113	1069
Pet allergy	72	932	65	1069
Spring allergy	270	728	71	1069
Allergy to other things	68	841	159	1068
chronic disease	Yes	No	Not sure	Total
diabetes	85	976	8	1069
hypertension	100	960	9	1069
coronary artery disease	64	995	10	1069
liver disease	8	1053	8	1069
kidney disease	9	1055	5	1069
immunodeficiency	10	1052	7	1069
hematological disease	15	1047	7	1069
endocrine disease	42	1016	11	1069
respiratory disease	41	1019	9	1069
neurological disease	8	1055	6	1069
other diseases	41	991	36	1068
Medications	Yes	No	Not sure	Total
Hypertension medications	103	963	3	1069
Diabetes medications	89	977	3	1069
Anticoagulants	95	971	3	1069
Asthma medications	37	1028	4	1069
Thyroid hormone	47	1016	6	1069
Analgesics (NSAIDs, Paracetam)	197	855	17	1069
Opioids	9	1056	4	1069
Immunosuppressants	22	1044	3	1069
Antidepressants	16	1048	5	1069
Antibiotics	65	997	7	1069
Contraceptives	20	1047	2	1069
Seizure medications	0	1067	2	1069
Others	74	983	11	1068

**Table 3:** Side effects after each vaccine injection throughout the entire course of vaccination.

	One Dose (*n* = 71) ^a^	Two Doses (*n* = 741) ^b^		Three Doses, (*n* = 257) ^c^		
	Single Dose	1st Shot	2nd Shot	1st Shot	2nd Shot	3rd Shot
Ageusia	0 (0%)	22 (3%)	37 (5%)	3 (1%)	3 (1%)	13 (5%)
Anosmia	2 (3%)	30 (4%)	44 (6%)	5 (2%)	23 (9%)	13 (5%)
Arthralgia	20 (28%)	348 (47%)	319 (43%)	54 (21%)	62 (24%)	105 (41%)
Chest pain	9 (13%)	89 (12%)	126 (17%)	26 (10%)	18 (7%)	28 (11%)
Chills	24 (34%)	348 (47%)	348 (47%)	82 (32%)	75 (29%)	90 (35%)
Cough	4 (6%)	74 (10%)	96 (13%)	31 (12%)	18 (7%)	26 (10%)
Diarrhea	6% (4)	44 (6%)	59 (8%)	10 (4%)	5 (2%)	8 (3%)
dizziness	11 (15%)	177 (24%)	185 (25%)	41 (16%)	15 (6%)	59 (23%)
Face/lips swelling	2 (3%)	22 (3%)	22 (3%)	3 (1%)	3 (1%)	3 (1%)
Fatigue	49 (69%)	585 (79%)	578 (78%)	157 (61%)	162 (63%)	167 (65%)
Fever	29 (41%)	358 (52%)	400 (54%)	93 (36%)	105 (41%)	116 (45%)
Headache	35 (49%)	489 (66%)	459 (62%)	100 (39%)	105 (41%)	131 (51%)
localized pain	53 (75%)	615 (83%)	585 (79%)	200 (78%)	167 (65%)	195 (76%)
localized Redness	24 (34%)	259 (35%)	274 (37%)	113 (44%)	89 (35%)	105 (41%)
Myalgia	27 (38%)	437 (59%)	393 (53%)	64 (25%)	67 (26%)	115 (45%)
Nasal congestion	13 (18%)	89 (12%)	104 (14%)	31 (12%)	21 (8%)	33 (13%)
Nausea	9 (13%)	126 (17%)	148 (20%)	15 (6%)	8 (3%)	21 (8%)
Neurological symptoms	0 (0%)	22 (3%)	30 (4%)	3 (1%)	5 (2%)	23 (9%)
rhinorrhea	13 (18%)	82 (11%)	104 (14%)	31 (12%)	23 (9%)	39 (15%)
Shortness of breath	16 (23%)	126 (17%)	163 (22%)	28 (11%)	15 (6%)	26 (10%)
Sneezing	6 (8%)	67 (9%)	93 (13%)	21 (8%)	13 (5%)	28 (11%)
Sore throat	11 (15%)	96 (13%)	89 (12%)	26 (10%)	21 (8%)	51 (20%)
Vomiting	6 (8%)	30 (4%)	37 (5%)	5 (2%)	3 (1%)	15 (6%)

^a^: Pfizer-BioNTech, *n* = 46 (64.8%), Sinopharm, *n* = 21 (29.6%), AstraZeneca, *n* = 4 (5.6%). ^b^: Pfizer-BioNTech, *n* = 410 (55.3%), Sinopharm, *n* = 303 (40.9%), AstraZeneca, *n* = 28 (3.8%). ^c^: Pfizer-BioNTech, *n* = 181 (70.4%), Sinopharm, *n* = 63 (24.5%), AstraZeneca, *n* = 13 (5.1%).

**Table 4:** Comparison of onset and duration of side effects after each injection of vaccine throughout the entire course of vaccination.

Variables	One Dose (*n* = 71) ^a^	Two Doses (*n* = 741) ^b^		X^2^ (*p*-Value) ^d^	Three Doses (*n* = 257) ^c^			X^2^ (*p*-Value) ^e^
	Single Dose	1st Shot	2nd Shot		1st Shot	2nd Shot	3rd Shot	
The Onset of Side Effects after Vaccination								
within 24 h	58 (82%)	615 (83%)	608 (82%)	0.09 (0.76)	231 (90%)	221 (86%)	244 (95%)	8.1 (0.045)
1–3 days	13 (18%)	111 (15%)	111 (15%)	0.17 (0.68)	26 (10%)	28 (11%)	10 (4%)	13.1 (0.0003)
3–5 days	0 (0%)	7 (1%)	7 (1%)	0.19 (0.65)	0 (0%)	8 (3%)	3 (1%)	2.7 (0.12)
1 week	0 (0%)	7 (1%)	15 (2%)	4.15 (0.042)	0 (0%)	0 (0%)	0 (0%)	Not Applicable
Duration of side effects								
less than 24 h	36 (51%)	207 28%)	81 (11%)	104 (<0.0001)	157 (61%)	177 (69%)	129 (50%)	1.3 (0.25)
1–3 days	29 (41%)	401 (54%)	363 (49%)	0.51 (0.48)	44 (17%)	59 (23%)	33 (13%)	13.9 (0.0002)
3–7 days	6 (8%)	89 (12%)	230 (31%)	81.2 (<0.0001)	44 (17%)	18 (7%)	59 (23%)	6.1 (0.015)
more than 1 week	0 (0%)	44 (6%)	67 (9%)	10.5 (0.0012)	12 (5%)	3 (1%)	36 (14%)	24.1 (<0.0001)

^a^: Pfizer-BioNTech, *n* = 46 (64.8%), Sinopharm, *n* = 21 (29.6%), AstraZeneca, *n* = 4 (5.6%). ^b^: Pfizer-BioNTech, *n* = 410 (55.3%), Sinopharm, *n* = 303 (40.9%), AstraZeneca, *n* = 28 (3.8%). ^c^: Pfizer-BioNTech, *n* = 181 (70.4%), Sinopharm, *n* = 63 (24.5%), AstraZeneca, *n* = 13 (5.1%). ^d^: X^2^ The Chi-squared test (*p*-value) for single dose shot to multi shots in two doses. ^e^: X^2^ The Chi-squared test (*p*-value) and for single dose shot to multi shots in three doses.

**Table 5:** The frequency of reported side effects and their association with different variables after each vaccine injection throughout the entire course of vaccination.

		One Dose (*n* = 71)		Two Doses (*n* = 741)				Three Doses (*n* = 257)					
		Single Dose		1st Shot		2nd Shot		1st Shot		2nd Shot		3rd Shot	
Variable	category	Reported Side Effects n (%)	P ^a^ (Phi Value) ^b^	Reported Side Effects n (%)	P ^a^ (Phi Value) ^b^	Reported Side Effects n (%)	P ^a^ (Phi Value) ^b^	Reported Side Effects n (%)	P ^a^ (Phi Value)^b^	Reported Side Effects n (%)	P ^a^ (Phi Value) ^b^	Reported Side Effects n (%)	P ^a^ (Phi Value) ^b^
Gender	Female	19 (59.4)	0.15	204 (57.6)	0.001, (0.53)	160 (58)	0.001, (0.38)	77 (63.6)	0.045 (0.13)	59 (60.8)	0.46	105 (60)	0.29
	Male	13 (40.6)		150 (42.2)		116 (42)		44 (36.4)		38 (39.2)		70 (40)	
Age	<18	14 (43.8)	0.9	55 (15.5)	0.9	56 (20.3)	0.006 (0.11)	Not Applicable					
	18 -40	18 (56.3)		299 (84.5)		220 (79.7)							
Smoker	No	31 (96.9)	0.35	311 (87.9)	0.041, (0.08)	244 (88.4)	0.042 (0.08)	84 (69.4)	0.8	66 (68)	0.75	121 (69.1)	0.84
	Yes	1 (3.1)		43 (12.1)		32 (11.6)		37 (30.6)		31 (32)		54 (30.9)	
Vaccine type	Pfizer	22 (68.8)	0.22	229 (64.7)	<0.001 (0.26)	195 (70.7)	<0.001 (0.27)	93 (76.9)	0.002 (0.25)	76 (78.4)	<0.001 (0.28)	6 (3.4)	<0.001 (0.33)
	Sinopharm	7 (21.9)		102 (28.8)		67 (24.3)		19 (15.7)		15 (15.5)		52 (29.7)	
	AstraZeneca	3 (9.4)		23 (6.5)		14 (5.1)		9 (7.4)		6 (6.2)		117 (66.9)	

^a^ *p*-value for the chi-squared test (X^2^) for the association. Significant level <0.05. ^b^ Phi factor was illustrated in cases of significant *p*-value to demonstrate the strength of the association.

## Data Availability

The data presented in this study are available on request from the corresponding author.
